# Endothelial Nitric Oxide Suppresses Action-Potential-Like Transient Spikes and Vasospasm in Small Resistance Arteries

**DOI:** 10.1161/HYPERTENSIONAHA.120.15491

**Published:** 2020-07-27

**Authors:** Josh F. Smith, Hamish A.L. Lemmey, Lyudmyla Borysova, C. Robin Hiley, Kim A. Dora, Christopher J. Garland

**Affiliations:** 1 From the Deptartment of Pharmacology, University of Cambridge (C.R.H.); 2 Department of Pharmacology, Universityxs of Oxford (J.F.S., H.A.L.L., L.B, K.A.D., C.J.G.).

**Keywords:** cardiovascular diseases, endothelium, nitric oxide, rats, vasoconstriction, vasospasm

## Abstract

Endothelial dysfunction in small arteries is a ubiquitous, early feature of cardiovascular disease, including hypertension. Dysfunction reflects reduced bioavailability of endothelium-derived nitric oxide (NO) and depressed endothelium-dependent hyperpolarization that enhances vasoreactivity. We measured smooth muscle membrane potential and tension, smooth muscle calcium, and used real-time quantitative polymerase chain reaction in small arteries and isolated tubes of endothelium to investigate how dysfunction enhances vasoreactivity. Rat nonmyogenic mesenteric resistance arteries developed vasomotion to micromolar phenylephrine (α_1_-adrenoceptor agonist); symmetrical vasoconstrictor oscillations mediated by L-type voltage-gated Ca^2+^ channels (VGCCs). Inhibiting NO synthesis abolished vasomotion so nanomolar phenylephrine now stimulated rapid, transient depolarizing spikes in the smooth muscle associated with chaotic vasomotion/vasospasm. Endothelium-dependent hyperpolarization block also enabled phenylephrine-vasospasm but without spikes or chaotic vasomotion. Depolarizing spikes were Ca^2+^-based and abolished by either T-type or L-type VGCCs blockers with depressed vasoconstriction. Removing NO also enabled transient spikes/vasoconstriction to Bay K-8644 (L-type VGCC activator). However, these were abolished by the L-type VGCC blocker nifedipine but not T-type VGCC block. Phenylephrine also initiated T-type VGCC-transient spikes and enhanced vasoconstriction after NO loss in nonmyogenic arteries from spontaneously hypertensive rats. In contrast to mesenteric arteries, myogenic coronary arteries displayed transient spikes and further vasoconstriction spontaneously on loss of NO. T-type VGCC block abolished these spikes and additional vasoconstriction but not myogenic tone. Therefore, in myogenic and nonmyogenic small arteries, reduced NO bioavailability engages T-type VGCCs, triggering transient depolarizing spikes in normally quiescent vascular smooth muscle to cause vasospasm. T-type block may offer a means to suppress vasospasm without inhibiting myogenic tone mediated by L-type VGCCs.

## Abstract 2

Supplemental Digital Content is available in the text.

Cardiovascular disease, including hypertension, is associated with a dysfunctional microcirculation including loss of vasomotion. Vasomotion occurs in arteries from a wide range of species, including humans, and sustains organ and tissue function by optimizing blood flow.^1–3^ It manifests as symmetrical, depolarizing oscillations of arterial smooth muscle membrane potential, underpinned by L-type voltage-gated calcium channel (VGCC) activity, that drives *circa* 0.05 to 0.2 Hz cycles of vasoconstriction/vasodilation. Vasomotion requires effective signaling cross-talk between arterial smooth muscle and endothelial cells and is abolished by compromised endothelial cell function, particularly the loss of NO-cGMP signaling.^2^

Although vasomotion is a widespread phenomenon involving depolarization, arterial smooth muscle cells are electrically quiescent and do not normally develop action potential-like spikes. Vasomotion appears during vasoconstriction stimulated either by constrictor agonists or, in myogenically active arteries, by an increase in intraluminal pressure. In both types of small artery, vasoconstriction is initiated by slow, graded smooth muscle depolarization that increases the open probability of L-type voltage-gated Ca^2+^ channels (VGCCs, Ca_V_1.2 α-subunit) allowing extracellular Ca^2+^ influx.^4,5^ Reports of transient spikes in arterial smooth muscle are restricted mainly to cerebral arteries and specifically cerebral arteries under abnormal conditions. For example, although the majority of human isolated cerebral arteries were electrically quiescent, transient spikes, referred to as action potentials, occurred in a small proportion of the arteries. The smooth muscle resting potential in the latter was quite depolarized, indicative of damage to the endothelium.^6^ Similar action-potential-like spikes were also recorded in cerebral arteries exposed to high intraluminal pressure (>100 mmHg), but in contrast this pressure did not generate action potentials in similar size peripheral resistance arteries.^5,7^ Overall, the electrophysiological characteristics of cerebral arteries therefore seem to differ from small peripheral arteries, and in a way that predisposes them to electrical excitability.^7^

The endothelium inhibits arterial smooth muscle electrical excitability by generating hyperpolarizing current to suppress or reverse depolarization. If vasomotion occurs, reversing depolarization creates the cycles of muscle depolarization/repolarization that drive oscillations in artery diameter. The mechanism depends on heterocellular signaling back and forth between the smooth muscle and endothelial cells. As smooth muscle [Ca^2+^]_i_ is raised causing vasoconstriction, Ca^2+^ and IP_3_ also pass to the endothelium via myoendothelial gap junctions. This signaling activates endothelial K_Ca_ channels, giving rise to hyperpolarization (endothelium-dependent hyperpolarization [EDH]), and stimulates nitric oxide (NO) synthase generating NO. These inhibitory signals then act on the smooth muscle to reverse or suppress vasoconstriction.^8–11^

Physiological vasomotion requires coordinated control of arterial smooth muscle electrical excitability. The present study investigated how loss of NO and EDH, as occurs in cardiovascular disease, impacts on the electrical activity of the vascular smooth muscle. As endothelial cell dysfunction is a ubiquitous early feature of cardiovascular disease, any change is of widespread relevance in understanding and addressing the associated increase in vasoreactivity and appearance of vasospasm.^12^

The current study used small nonmyogenic mesenteric arteries, including spontaneously hypertensive rat (SHR) arteries, and myogenically active intraseptal coronary arteries. As expected, loss of either endothelial NO or EDH increased vasoreactivity and disrupted vasomotion. However, loss of NO dramatically altered smooth muscle electrical activity, by enabling the appearance of Ca^2+^-based transient spikes associated with vasospasm. The use of selective blockers suggested this change reflects an essential de novo input by T-type VGCCs to trigger the spikes, which when triggered also encompassed input from L-type VGCCs. A similar profile was observed in SHR arteries. In contrast to the loss of NO, loss of EDH did not enable transient spikes. However, it did abolish the transient hyperpolarization/vasorelaxation observed during chaotic vasomotion stimulated by NO loss. In myogenic coronary arteries, transient spikes were also enabled by loss of NO, with block of these events reversing the associated vasospasm but not myogenic tone.

## Methods

Rat small mesenteric and coronary arteries were isolated and mounted in a Mulvany-Halpern myograph for simultaneous measurement of tension and either membrane potential and intracellular calcium. Isolated arteries and endothelial cell tubes were used for real-time polymerase chain reaction.^11,13–15^ Data were analyzed using Microsoft Excel 2011 (Microsoft Corporation) and GraphPad Prism (v8.0, GraphPad Software) software. Details can be found in the Data Supplement, Expanded Materials and Methods. Data that support the findings of this study are available from the corresponding author upon reasonable request.

## Results

### Smooth Muscle Transient Depolarizing Spikes Enabled By Loss of Endothelial NO But Not EDH

**Figure 1.**
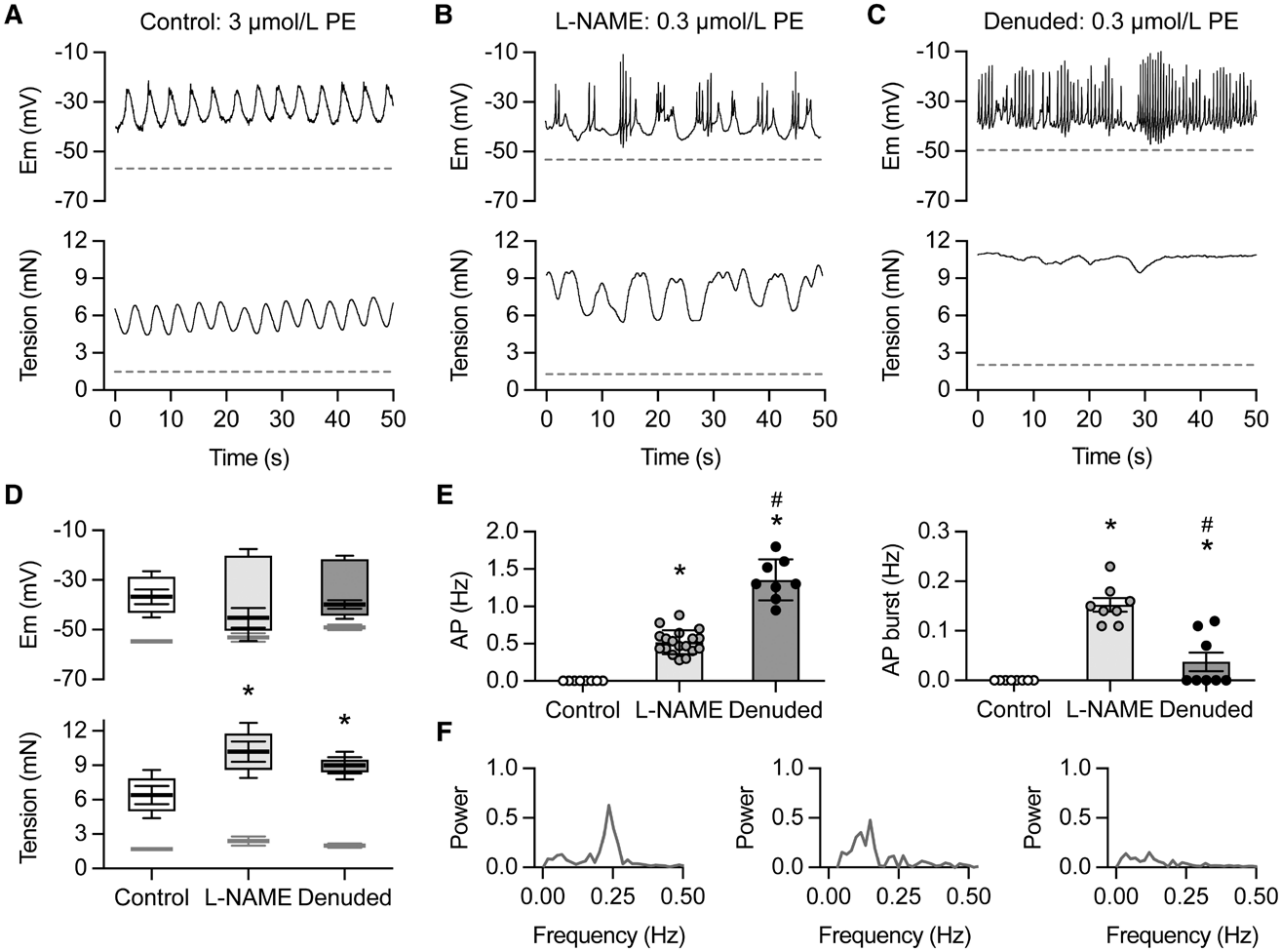
Removing nitric oxide (NO) converts physiological vasomotion into rapid depolarizing spikes and vasospasm. **A**, Vasomotion to 3 µM phenylephrine (PE). **B**, After block of endothelial NO synthase, 10-fold less PE caused a chaotic vasomotion associated with bursts of transient spikes and enhanced vasoconstriction. **C**, In endothelium denuded arteries, 0.3 µmol/L PE stimulated continuous transient spike firing and tetanic vasospasm. Dotted lines in **A–C** represent membrane potential and tension before exposure to PE. **D**, Box-plots summarize the amplitude of vasomotion, depolarizing spikes and vasoconstriction as mean±SEM for the maximum and minimum values of mV and mN with sample mean. Membrane potential and tension before (light gray) and in the presence of 3 µmol/L PE in control (open box, n=6), and 0.3 µmol/L PE with L-NAME (light gray box, n=18) and denuded arteries (darker gray box, n=8). Difference from control *P<0.05. **E**, Left, denuded arteries displayed greater transient spike frequency, 1.3±0.1 Hz, than endothelium-intact arteries with L-NAME, 0.72±0.07 (#*P*<0.05); both greater than control (vasomotion, 0.23±0.01 Hz) *P<0.001. Right, frequency of transient spike bursting greatest with L-NAME compared with control or denuded arteries (#L-NAME/denuded difference, *P*<0.05). Both differed from control (*P<<0.001 and <0.05). **F**, Fourier transform shows the primary waveform for vasoconstriction/vasorelaxation, from left, during vasomotion (0.22 Hz mean of 6 arteries to 3 µmol/L), equivalent in arteries exposed to L-NAME with chaotic vasomotion (0.13 Hz, n=8–0.3 µmol/L PE) and denuded arteries developing vasospasm without oscillation (no overriding frequency, n=8 to 0.3 µmol/L PE).

The α_1_-adrenoreceptor agonist, phenylephrine (PE, 0.1–5 µmol/L) stimulated VSM depolarization and, from *circa* 1 µmol/L, vasomotion (Figure 1A). Reducing endothelial vasodilator capacity with 100 μmol/L L-NAME (Figure 1B) or by physically denuding (stripping off) endothelial cells (Figure 1C), abolished vasomotion so less phenylephrine now initiated rapid, transient depolarizing spikes and greater vasoconstriction. Figure 1A shows phenylephrine -evoked vasomotion to 1 µmol/L and Figure 1B and 1C similar depolarization to 0.3 µmol/L with L-NAME or after denudation (Figure 1B and 1C) causing transient spikes and greater vasoconstriction.

With 100 μmol/L L-NAME, phenylephrine initiated bursts of transient depolarizing smooth muscle spikes, similar to action potentials, interspersed by periods of repolarization and partial vasorelaxation. This switched vasomotion into a chaotic waveform (Figure 1B, 1D through 1F). Spike frequency was greater in the additional presence of 0.1 µmol/L paxilline to block BK_Ca_ channels (from 0.72±0.07, n=18 to 1.4±0.1 Hz, *P*<0.001, n=5), although paxilline alone did not initiate spikes. Depolarizing spikes are shown in high resolution in Figure S1 in the Data Supplement and enhanced vasoreactivity in Figure S2A.

Endothelial denudation also dramatically raised vasoreactivity, but the transient spikes fired continuously, resulting in sustained tetanic vasospasm without intermittent vasorelaxation (Figure 1C through 1F). With both L-NAME and denudation, transient smooth muscle spikes occurred once phenylephrine -depolarization reached *circa* -40mV mV (from resting potentials of −53.9±1.7 or −49.8±1.2 mV, respectively), while in control arteries spikes did not occur even with the highest phenylephrine concentration. Box-plots are used in Figure 1 to summarize the relative amplitudes of vasomotion illustrated in Figure 1A or depolarizing spikes in Figure 1B and 1C.

**Figure 2.**
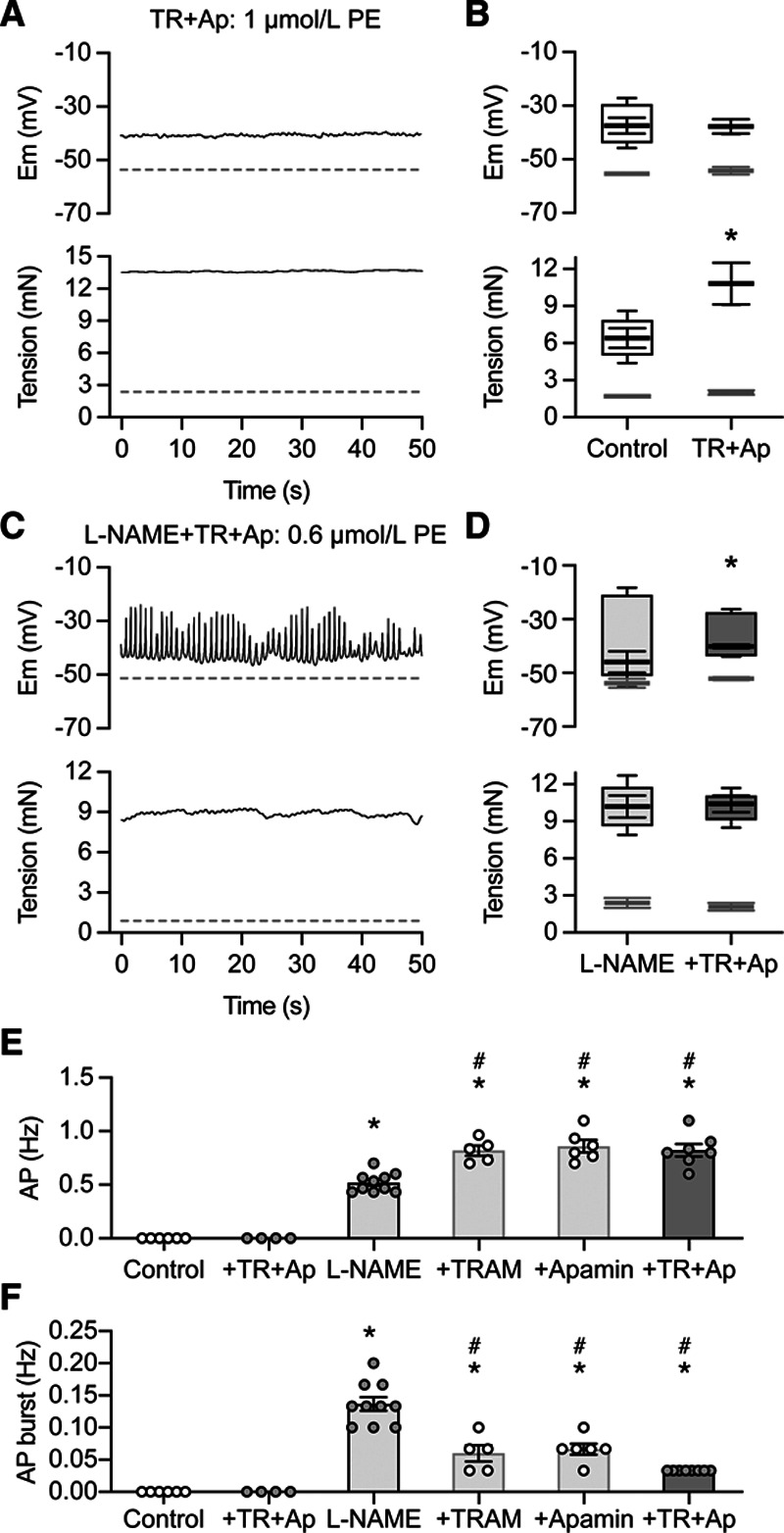
Loss of endothelium-dependent hyperpolarization does not enable transient spikes. **A**, Vasoconstriction to 1 µmol/L phenylephrine (PE) was enhanced by blocking endothelium-dependent hyperpolarization (EDH) with 100 nmol/L apamin and 1 µmol/L TRAM-34, but although vasomotion was lost transient spikes were not triggered, although the smooth muscle depolarized to −40 mV **B**, Membrane potential and tension before (light gray) and in the presence of 3 µmol/L PE (control vasomotion, open box-plot; n=6), and 1 µmol/L PE with apamin and TRAM-34 present (n=6). Box-plots show mean±SEM for the maximum and minimum values of mV and mN. Mean depolarization (box-plot) to PE did not differ between groups but vasomotion was abolished and vasoconstriction enhanced (*P<0.05). **C**, Additional block of NO synthase led to continuous transient spike firing and vasospasm with PE (with 100 µmol/L L-NAME, 0.1 µmol/L apamin, and 1 µmol/L TRAM-34, responses similar to denuded arteries, Figure 1C). Dotted lines in (**A**) and (**C**) show membrane potential and tension before exposure to PE. **D**, Mean±SEM membrane potential and tension before (light gray box-plot) and in the presence of 0.6 µmol/L PE in each case (darker gray box-plot, n=7). The addition of TRAM-34 and apamin (dark gray) reduced the transient spike amplitude to PE (*P<0.05) but not vasospasm (*P*>0.05). **E**, PE did not evoke transient spikes in control arteries or with TRAM-34 and apamin present. Once L-NAME was present, PE evoked transient spikes and frequency was enhanced by blocking EDH. *Each different to control and to TRAM-34 and apamin *P*<0.0001. #Higher frequency than L-NAME alone, *P*<0.001. **F**, Transient spike burst frequency with L-NAME was supressed by the further addition of either TRAM-34, apamin, or the 2 in combination, *P<0.01 in each case. In combination with L-NAME, TRAM-34 and apamin reduced bursting more than apamin or TRAM-34 alone #*P*<0.05.

Vasoreactivity to phenylephrine was also raised by blocking EDH, a mechanism that provides significant vasodilator input alongside NO in small arteries (Figure 2A; Figure S2B). EDH was blocked with 100 nmol/L apamin (blocks K_Ca_2.3/SK_Ca_) and 1 µmol/L TRAM-34 (blocks K_Ca_3.1/IK_Ca_). In contrast to loss of NO, EDH block did not initiate transient spikes, although the smooth muscle depolarized beyond −40 mV with phenylephrine (Figure 2A and 2B). However, EDH block abolished vasomotion and enhanced vasoconstriction to phenylephrine. Subsequent inhibition of NO synthase in the same arteries now enabled transient spikes (Figure 2C and 2D). These spikes and associated vasospasm were similar to the response of denuded arteries (compare Figure 2E and 2F with Figure 1E and 1F). Therefore, intermittent repolarization and vasorelaxation recorded in the presence of L-NAME alone may be explained by indirect activation of EDH-vasodilation by phenylephrine, following increased smooth muscle Ca^2+^.^9,11^

### Smooth Muscle Transient Depolarizing Spikes Require T-Type VGCC Input

**Figure 3.**
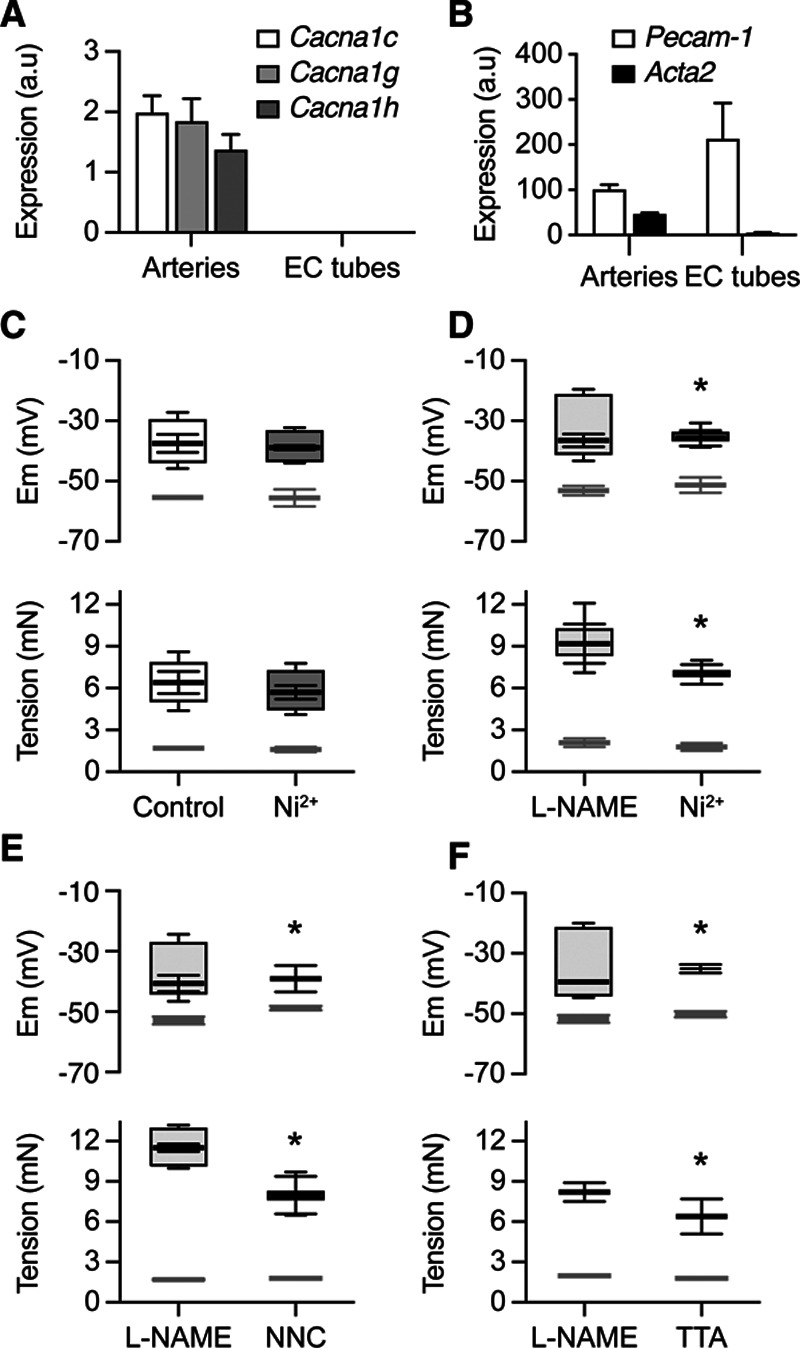
T-type voltage-gated Ca^2+^ channels (VGCCs) trigger transient spikes to phenylephrine (PE) **A**, Bar graphs summarize gene expression for L- and T-type Cav channel isoforms (Cav1.2, Cacna1c; Cav3.1, Cacna1g; and Ca_v_3.2, Cacna1h) in arterioles and in EC tubes lacking VSM. **B**, Gene expression for EC (PECAM-1, Pecam1) and VSM (α-smooth muscle actin, Acta2) markers in arteries and in EC tubes. Data shown as mean±SEM; n=4 sets of pooled mRNA samples from 4 animals, with the same source tissue used for (**A**) and (**B**). **C**, Mean±SEM membrane potential and tension before (light gray) and during vasomotion to 3 µmol/L PE (control, n=4), and in arteries preexposed to 50 µmol/L Ni^2+^ (n=6). Boxes show mean±SEM for the maximum and minimum values of mV and mN. Neither mean depolarization nor vasomotion amplitude to PE differed between groups (*P*>0.05) **D**, Mean±SEM membrane potential and tension before (light gray) and then transient spikes and vasoconstriction to 0.5 µmol/L PE in the presence of L-NAME. Boxes show maximum and minimum mV and mN. Subsequent exposure to 1 µmol/L PE but with 50 µmol/L Ni^2+^ present caused similar depolarization but without transient spikes and with reduced vasoconstriction, *P<0.05 mN, compared with L-NAME alone, n=6. **E**, About 0.3 µmol/L NNC 55-0396 also blocked the appearance of transient spikes and reduced PE vasoconstriction *P<0.05 mV, n=5. Box-plots show control (L-NAME and 0.7 µmol/L PE). Similar depolarization was evoked with NNC 55-0396 (to 1 µmol/L PE) but without transient spikes and with reduced vasoconstriction. **F**, About 0.3 µmol/L TTA-A2 also blocked transient spikes to (0.6 µmol/L) PE after L-NAME. With TTA-A2 present, slightly more depolarization was evoked (#*P*<0.05), but without transient spikes and with less vasoconstriction (*P<0.05 in each case, n=5). **C–F**, Mean±SEM for resting membrane potential and tension before PE is shown in light-gray.

The smooth muscle spikes were dependent on de novo input from latent T-type VGCCs (Ca_v_3.x). A positive real-time quantitative polymerase chain reaction signal for 2 forms of T-type VGCCs, Ca_V_3.1, and Ca_V_3.2 (Cacna1g; and Cacna1h) was evident in intact mesenteric arteries alongside Ca_V_1.2 (Cacna1c, L-type VGCC), but isolated tubes of mesenteric endothelial cells failed to show any evidence of VGCCs, consistent with smooth muscle localization (Figure 3A and 3B). Functionally, phenylephrine vasomotion was not modified by the T-type VGCC blocker, 50 μmol/L Ni^2+^ (Figure 3C, Figure S3A), but in contrast transient phenylephrine-spikes after L-NAME were abolished and vasoconstriction reduced when Ni^2+^ was applied during spike firing (Figure S3B) or before stimulation with phenylephrine (Figure 3D; Figure S3C). At 50 µmol/L Ni^2+^ is sufficiently selective against T-type VGCCs to distinguish between Ca_V_3.1 and Ca_V_3.2, IC_50_ ≈250 and 12 µmol/L, respectively.^16^ Other structurally unrelated T-type VGCC blockers also inhibited transient spikes and vasoconstriction to phenylephrine; 0.3 µmol/L NNC 55-0396, a nonhydrolysable derivative of mibefradil (Figure 3E) or 0.3 µmol/L TTA-A2 (Figure 3F). In both cases, loss of transient spikes was accompanied by reduced vasoconstriction. NNC 55-0396 also reversed the increase in phenylephrine vasoconstriction after endothelial denudation but not the raised sensitivity that followed block of EDH (Figure S2). The concentration of T-type VGCC blockers used did not inhibit L-type VGCCs, as vasoconstriction to the specific channel activator Bay K-8644 was unaffected (Figures S4 and S5).

### Smooth Muscle Transient Spikes Are Ca^2+^ Based

**Figure 4.**
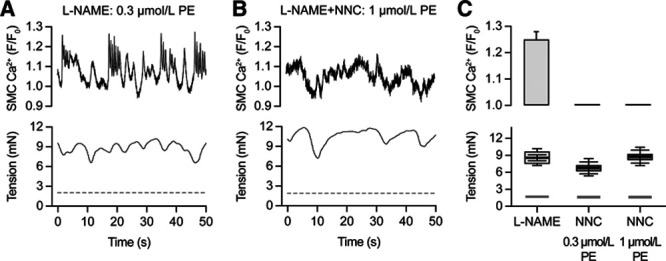
Intracellular Ca^2+^ flashes induced by phenylephrine (PE) in mesenteric artery smooth muscle sensitive to T-type voltage-gated Ca2+ channel (VGCC) block. **A**, About 0.3 µmol/L PE evoked Ca^2+^ flashes (upper) and chaotic vasomotion (lower) in arteries exposed to 100 µmol/L L-NAME. **B**, About 0.3 µmol/L NNC 55-0396 blocked the appearance of smooth muscle Ca^2+^ flashes even to 1 µmol/L PE. **C**, Summary data for Ca^2+^ flashes and tension change to 0.3 µmol/L PE in the presence of L-NAME (gray box) and to 0.3 and 1 µmol/L PE (middle and right) in the presence of 0.3 µmol/L NNC 55-0396. Boxes represent mean±SEM for Ca^2+^ and tension change and minimum and maximum response, n=5 separate arteries in each case. Prestimulation values are shown in light-gray.

Phenylephrine stimulated flashes of intracellular smooth muscle Ca^2+^ (Figure 4A) and with similar frequency (0.45±0.08 Hz, n=5) to transient spikes evoked in the combined presence of phenylephrine and L-NAME (0.72±0.07 Hz, *P*>0.05, n=18). Ca^2+^ flashes were blocked by 0.3 µmol/L NNC 55-0396 (Figure 4B and 4C), which did not alter Ca^2+^ flashes evoked by the L-type VGCC activator Bay-K 8644 (Figure S5).

### L-Type VGCCs Contribute to Firing of Transient Spikes Once NO Availability Is Reduced

Although T-type VGCCs were essential for spike firing, block was also apparent with the L-type (Ca_V_1.2) VGCC blocker 0.3 µmol/L nifedipine. Nifedipine blocked transient spikes induced with phenylephrine and partially reversed depolarization, by 9.3±0.6 mV (n=6). In denuded arteries preincubated with nifedipine, depolarization was still evoked with phenylephrine, but now without triggering spikes (Figure S5D and S5E). Greater phenylephrine concentrations were necessary for an equivalent depolarization in the presence of nifedipine.

Transient spikes could be induced by the L-type VGCC activator Bay-K 8644 but only once endothelial function had been compromised. In arteries with a functional endothelium, Bay-K 8644 did not stimulate depolarization (to 3 µmol/L, n=5), but in denuded arteries, transient spikes (10–30 nmol/L Bay-K 8644, threshold −46.7±1.6 mV, n=5) and associated vasoconstriction were induced (Figure S5A and S5B). Both transient spikes and associated vasoconstriction were insensitive to either NNC 55-0396 (Figure S5A) or TTA-A2 (Figure S5F), but abolished by nifedipine (Figure S5B). L-type VGCCs (Ca_V_1.2) underpin vasomotion, which is blocked with dihydropyridines such as nifedipine.^17^ Thus, although L-type VGCCs could generate smooth muscle transient spikes, they were not initiated during vasomotion (when endothelial NO is available), even though depolarization reached threshold values of <−40 mV.

### NO Suppresses Transient Spikes Via cGMP

**Figure 5.**
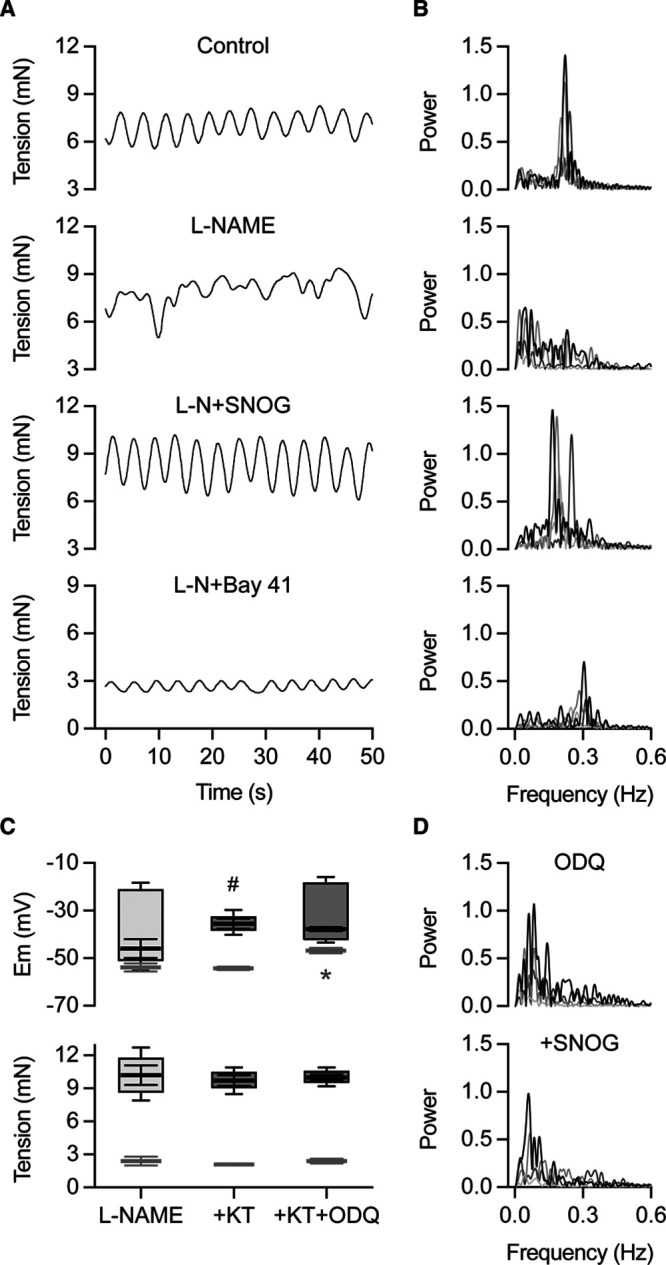
Nitric oxide (NO) supresses transient spikes by signalling through cGMP. **A**, Vasomotion (mN) to 3 µmol/L phenylephrine (PE) evolves into chaotic vasomotion once NO synthase is blocked with 100 µmol/L L-NAME (and now induced with only 0.3 µmol/L PE), reverting to vasomotion on addition of 2 µmol/L *S*-nitrosoglutathione (and requiring 3 µmol/L PE). The soluble guanylyl cyclase activator, 1 µmol/L Bay 41-2272 also converts chaotic vasomotion back to vasomotion in the presence of L-NAME and 3 µmol/L PE, n=5. **B**, These changes can be represented as prominent waveforms to PE extracted with Fourier analysis in each case. **C**, Mean±SEM membrane potential and tension before (light-gray, L-NAME) and during exposure to 0.3 µmol/L PE in the presence of the protein kinase G inhibitor 1 µmol/L KT 5823 (gray, +KT), and then including the soluble guanylyl cyclase inhibitor 10 µmol/L 1H-[1,2,4]oxadiazolo[4,3-a]quinoxalin-1-one (ODQ) (darker gray), n=5. Similar depolarization in the presence of L-NAME and KT 5823 (center box-plot) and L-NAME and KT 5823 together with ODQ (right box-plot) required 2 and 0.3 µmol/L PE, respectively. Transient spikes to PE only appeared once ODQ was present, apparent as increased mV amplitude box-plot with the latter, #*P*<0.001. Resting membrane potential (light-gray) was depolarized with ODQ present, * difference *P*<0.05. Tension with either 2 µmol/L PE (with KT 5823) or 0.3 µmol/L PE (KT 5823+ODQ) was not different (*P*>0.001). Equivalent responses to L-NAME and 0.3 µmol/L PE from Figure 1D are shown for comparison. **D**, About 2 µmol/L SNOG failed to re-establish vasomotion when chaotic vasomotion to PE was induced with 10 µmol/L ODQ present, n=11. ODQ indicates 1H-[1,2,4] oxadiazolo[4,3-a]quinoxalin-1-one; and SNOG, *S*-nitrosoglutathione.

The ability of NO to sustain vasomotion was probed by modifying various points in the cGMP signaling pathway. The NO-donor, S-nitrosoglutathione (SNOG) re-established vasomotion from the chaotic vasomotion that followed inhibition of NO-synthase (with L-NAME, Figure 5A). Vasomotion could also be restored by activating sGC (soluble guanylyl cyclase) directly with Bay 41-2272, albeit the cycles were of low amplitude (Figure 5A, bottom trace). Vasomotion with SNOG or Bay 41-2272 had similar frequency, 0.23±0.01 Hz and 0.22±0.01 Hz (n=5), with the predominant waveform in each case shown in Figure 5B.

Inhibiting sGC with ODQ mirrored the effect of L-NAME, as this caused transient spikes and chaotic vasomotion to phenylephrine (Figure 5C and 5D). In contrast, block of protein kinase G with KT 5823 did not modify phenylephrine-vasomotion (Figure 5C). SNOG failed to re-establish vasomotion from chaotic vasomotion caused by ODQ, indicating that signaling beyond sGC was necessary (Figure 5D).

### Block of T-Type VGCCs Inhibits Transient Spikes and Vasoconstriction in SHR Arteries

**Figure 6.**
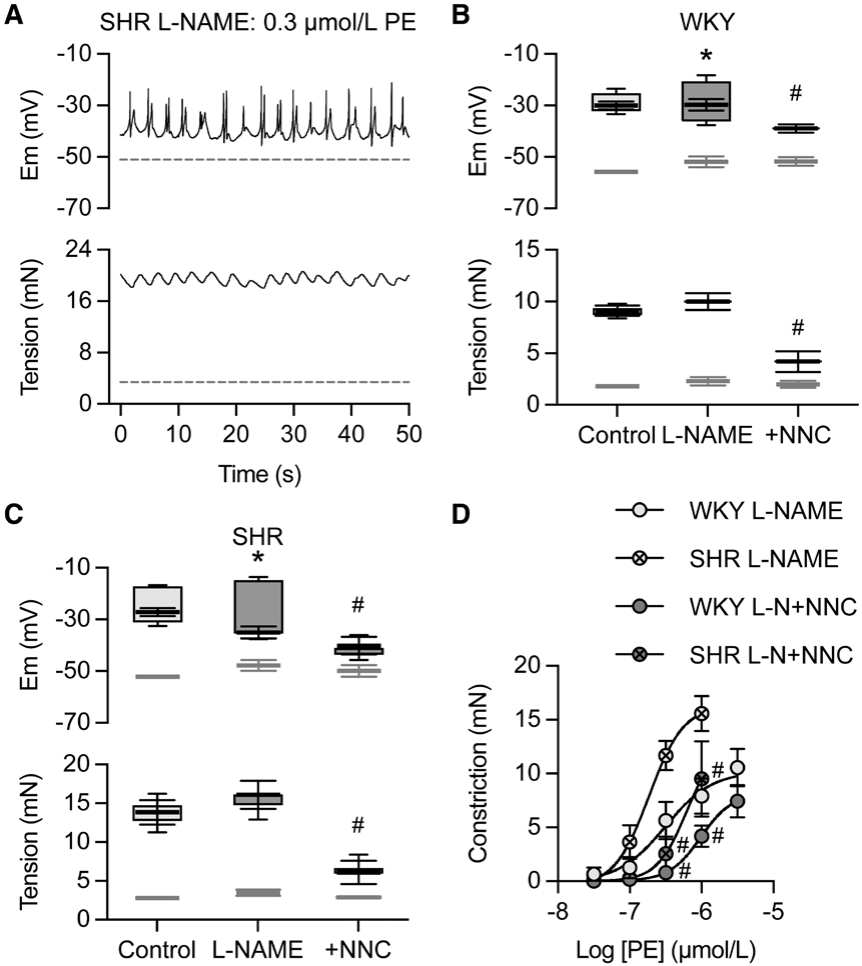
Phenylephrine (PE) induces transient spikes and vasospasm in spontaneously hypertensive rat mesenteric arteries sensitive to NNC 55-0396 after L-NAME. **A**, Representative trace from spontaneously hypertensive rat mesenteric artery showing transient spikes and vasospasm to 0.3 µmol/L PE in the presence of 100 µmol/L L-NAME. **B**, Summary of membrane potential and tension in WKY arteries before (light-gray) and in the presence of 1 µmol/L PE (control, open box-plot, n=6). The addition of 100 µmol/L L-NAME to the same artery increased sensitivity and enabled transient spikes to a lower PE concentration (mean data to 0.3 µmol/L PE showing increased amplitude, gray box-plot *P<0.05, n=6), which were abolished by 0.3 µmol/L NNC 55-0396, with reduction in both mean membrane potential and amplitude (to 1 µmol/L PE with L-NAME present, darker gray box-plot, n=5, *P*<<0.05 and <0.001, respectively) and greatly reduced vasoconstriction (*P*<0.0001), # changes compared with L-NAME alone. **C**, Summary of membrane potential and tension in spontaneously hypertensive rat arteries before (light-gray) and in the presence of 1 µmol/L PE (control, open box-plot, n=5). With 100 µmol/L L-NAME, similar depolarization followed lower PE concentration but now with transient spikes (0.3 µmol/L, gray box-plot, amplitude increase *P<0.05, n=5). Transient spikes were abolished in the presence of 0.3 µmol/L NNC 55-0396 (indicated by amplitude but not mean membrane potential reduction to 0.3–1 µmol/L PE plus L-NAME, *P*<0.0001) and reduced mean vasoconstriction (*P*<0.0001). # changes compared with L-NAME alone. In each case, box-plots display mean±SEM of maximum and minimum mV and mN. **D**, Summary of WKY (open circles) and spontaneously hypertensive rat (crosses) artery vasoconstriction to cumulative PE addition in the presence of L-NAME. About 0.3 µmol/L NNC 55-0396 markedly depressed vasoconstriction in each case, filled symbols, #*P*<0.01, n=5. SHR indicates spontaneously hypertensive rat.

As with Wistar and WKY arteries, phenylephrine also stimulated vasomotion in SHR mesenteric arteries that was abolished by blocking NO synthase with L-NAME, to be replaced by transient depolarizing spikes Figure 6A through 6C. Transient spikes were sensitive to block with NNC 55-0396 (Figure 6C), accompanied by marked attenuation of vasoconstriction Figure 6C and 6D. The dramatic decline in vasoconstriction reflected the greater vasoconstriction to phenylephrine in SHR arteries (compared with WKY/Wistar Figure S6).

### Myogenic Tone Persists After Block of Coronary Artery Transient Spikes

Intraseptal coronary arteries spontaneously developed myogenic tone associated with smooth muscle depolarization. L-NAME block of NO synthase caused further depolarization and vasoconstriction associated with the appearance of transient depolarizing spikes sensitive to 0.3 µmol/L NNC 55-0396, which also reversed the additional L-NAME evoked vasoconstriction (Figure S7).

## Discussion

The main finding is that the quiescent vascular smooth muscle cells in small resistance arteries generate transient depolarizing spikes on loss of NO. This switch in electrical excitability alters vasoreactivity so that physiological vasomotion disappears and small artery vasospasm develops. The fact this happens in both myogenic and nonmyogenic resistance arteries suggests it is characteristic of the smooth muscle in resistance arteries and as such of direct relevance in cardiovascular disease where endothelial dysfunction is a ubiquitous feature. In terms of agonist stimulation, in preliminary experiments, we observe similar spike firing with the thromboxane receptor agonist, U46619, in the absence of NO, suggesting the mechanism is shared by vasoconstrictor agonists. To trigger smooth muscle transient spikes, input from latent T-type VGCCs appears to be essential; the L-type VGCCs responsible for vasomotion were not sufficient. Identifying a central role for T-type VGCCs in small artery vasospasm offers a novel therapeutic target to oppose dysfunction in the microcirculation. An additional advantage of T-type rather than L-type VGCC block is that myogenic tone will be protected. Myogenic tone is mediated by L-type VGCCs and underpins tissue blood flow autoregulation with loss causing local tissue oedema, complicating the use of L-type VGCC blockers.

Apart from the portal vein,^18^ vascular smooth muscle cells do not usually generate rapid, transient depolarizing spikes. Stimulation with vasoconstrictor agents, or in myogenic arteries moderate changes in stretch reflecting intraluminal pressure, simply depolarize the vascular smooth muscle, increasing L-type VGCC (Ca_V_1.2) open probability, Ca^2+^ influx and vasoconstriction.^4^ The electrical quiescence of vascular smooth muscle cells seems largely due to a stabilizing influence by Ca^2+^-activated potassium channels (K_Ca_), as action-potential-like depolarizing spikes and associated vasoconstriction develop once these channels are inhibited.^19–21^ However, this is clearly not the only attenuating influence, as we show both myogenic and nonmyogenic arteries generate transient depolarizing spikes when BK_Ca_ channels are available, although providing an inhibitory input as demonstrated by the increased frequency of transient spikes with paxilline present. However, the ability of endothelial NO to suppress input by smooth muscle T-type VGCCs seems to be a major mechanism influencing artery function.

Although L-type VGCCs underpin the vasomotion that develops when the endothelium is able to generate NO, depolarization underpinning the symmetrical vasoconstrictor oscillations did not trigger transient spikes, although the membrane potential declined beyond the spike threshold of around −40 mV with high concentrations of phenylephrine. However, once NO synthesis was prevented, L-type VGCCs were able to initiate transient spikes on direct activation with Bay-K 8644. As expected, nifedipine blocked the transient spikes to Bay-K 8644 and they were resistant to T-type blockers in concentrations that blocked the transient spikes to phenylephrine, or in coronary arteries the loss of NO. However, the fact that the latter events were sensitive to nifedipine indicates that L-type VGCCs can contribute to but do not initiate spike firing. That Bay-K 8644 was only able to induce transient spikes in the absence of NO might be explained by an increased L-type VGCC conductance.^22^ Whatever the explanation, T-type VGCCs are essential to trigger transient spikes, an observation that is consistent with neurones, where T-type VGCC current can boost weak depolarization in dendrites to enable action potential firing.^23,24^ The facilitation is thought to reflect T-type VGCC kinetics, in particular, the relatively low voltage threshold and ability to generate window currents that enhance [Ca^2+^]_i_. T-type VGCC window currents in arterial smooth muscle peak at around −40 mV, which aligns with the transient spikes threshold recorded in the present study.^16,25,26^

In contrast to NO, block of EDH with apamin and TRAM-34 failed to enable transient spike firing, despite the fact that phenylephrine vasomotion was abolished and vasoconstriction enhanced. In the absence of EDH, but continued presence of NO, depolarizing, transient spikes did not occur even though phenylephrine reduced the smooth muscle membrane potential beyond −40 mV. However, subsequent block of NO synthase did enable spikes in these arteries, and with a continuous firing profile similar to denuded arteries. This contrasted with arteries in which EDH was available, where transient spikes and vasospasm were interspersed by brief hyperpolarization/vasorelaxation creating a chaotic, nonfused vasospasm. The intermittent profile of transient spikes reflects intercellular Ca^2+^ signaling from the smooth muscle to the endothelium. The Ca^2+^ signal activates the endothelial K_Ca_ channels responsible for EDH, providing inhibitory (hyperpolarizing) feedback to the smooth muscle.^11^ Boosting EDH when the endothelium is at least partially sound might provide a way to limit or reverse vasospasm in the microcirculation. In small arteries, EDH is a major functional influence, because the density of smooth muscle VGCCs, and thus sensitivity to membrane potential change, is inversely related to artery size.^27,28^

Demonstrating a critical role for T-type VGCCs relied on the use of selective concentrations of pharmacological blockers. Low micromolar Ni^2+^ blocks T-type VGCCs selectively in a range of cells, including cardiac and vascular smooth muscle cells, and differentiates between Ca_V_3.1 (IC_50_ circa 250 µmol/L) and Ca_V_3.2 (IC_50_ circa 12 µmol/L).^16,29^ In the present study, 50 µmol/L Ni^2+^ did not alter transient spikes or vasoconstriction evoked by the Ca_V_1.2 activator, Bay-K 8644, and its ability to block phenylephrine-induced spikes was similar to 2 other structurally unrelated T-type blockers, the nonhydrolysable derivative of mibefradil NNC 55-0396 and the structurally unrelated T-type VGCC blocker, TTA-A2.^30–32^ NNC 55-0396 has been reported to inhibit VSM K_V_ channels at positive membrane potentials, but this is not of concern as we were focussed on a negative range of potentials and blocking K_V_ would cause depolarization and vasoconstriction, the opposite to our observation.^33^ Overall, our data from both mesenteric and coronary small arteries are therefore consistent in indicating a crucial role for T-type VGCCs in triggering transient spikes and associated vasoconstriction. A sensitivity to 50 µmol/L Ni^2+^ suggests Ca_V_3.2 VGCCs may be the predominant channel.

The fact that block of NO synthase with L-NAME-initiated spike firing suggested signaling via cGMP normally suppresses this mechanism. This idea was supported by the demonstration that ODQ, which blocks sGC had a similar effect to L-NAME, it enabled transient spikes. Furthermore, in the presence of L-NAME, vasomotion could be re-established by adding NO using the NO-donor, SNOG, or by direct activation of sGC with Bay 41-2272. Interestingly, block of PKG did not cause transient spikes, suggesting cGMP may be acting directly to suppress T-type VGCCs in some way. This contrasts with cerebral artery smooth muscle where cGMP signaling via PKG does seem to be important, as direct activation of this enzyme suppressed T-type currents in isolated smooth muscle cells.^34^ However, the interaction between NO and T-type VGCCs is clearly complex. In neurones, NO acts to suppress T-type VGCC-based action potentials directly and via sGC/cGMP/PKG signaling.^35,36^ Recruiting T-type VGCCs might also, at least in part, involve channel recruitment to the surface membrane, as there is limited evidence to suggest Ca_V_3.1 moves to the surface membrane on loss of NO.^37^ How this occurs and whether it is a general mechanism is not clear.

In hypertension, endothelial dysfunction is associated with a reduction in both NO release and EDH,^12,38^ raising the possibility that in this disease the microcirculation might be prone to develop action potentials and vasospasm. However, the electrophysiological profile of SHR mesenteric arteries was similar to control vessels, with normal vasomotion during phenylephrine stimulation and T-type VGCC-dependent transient spikes appearing once NO synthase was blocked. SHR arteries did develop much greater vasoconstriction, presumably because of the thicker vessel wall.^39,40^ As a result, block of T-type VGCCs had a very large inhibitory effect against vasoconstriction. Greater vasoconstriction also minimized intermittent vasorelaxation. The animals used were between 3 and 4 months of age, so future studies are needed to show if older arteries develop spontaneous transient spikes as NO loss progresses.

Small (intraseptal) coronary arteries also developed T-type VGCC transient spikes, but in contrast to mesenteric arteries without the need for vasoconstrictor stimulation. Only block of NO synthase. The spontaneous appearance of transient spikes on loss of NO is similar to myogenic cerebral arteries, so this seems not to be a peculiarity of the brain vessels.^41^ In coronary arteries, although block of T-type VGCCs abolished transient spikes and associated vasoconstriction, preL-NAME myogenic tone remained. This raises the possibility that T-type blockers might be used to reverse the enhanced vasoconstriction/vasospasm caused by cardiovascular disease-linked endothelial dysfunction, while retaining myogenic reactivity to support blood flow autoregulation. In the heart, this may then protect against compromised coronary flow reserve. Although T-type VGCCs have previously been suggested to contribute to myogenic tone in some small arteries even when NO is available, that is, cerebral, renal, mesenteric, and cremaster arterioles, in each case, the contribution to function was very small and seemed only relevant at low intraluminal pressure, when the smooth muscle membrane potential is large and within the optimal voltage range for T-type VGCCs.^42–44^

### Perspectives

We have shown that compromising endothelial NO bioavailability in both myogenic and nonmyogenic resistance arteries switches vascular smooth muscle cells into an electrically excitable state by engaging T-type VGCC that trigger Ca^2+^ based transient spikes leading to vasospasm. As well as causing vasoconstriction, Ca^2+^ signals passing from the smooth muscle to the endothelium can provide negative feedback by activating EDH to abrogate vasospasm by brief, intermittent phases of vasorelaxation. This mechanism is summarized in the flow chart in Figure S8. As reduced NO bioavailability is a common feature of cardiovascular disease, these data suggest that block of T-type VGCCs may offer a novel way to oppose small artery vasospasm. In the microcirculation, loss of endothelial vasodilator capacity is known to precede and predict conduit artery pathology,^45,46^ and in the coronary microcirculation, this is reflected in reduced coronary flow reserve and angina-like chest pains in patients without obstructive disease.^47^ In some of these patients, T-type VGCCs blockers have been shown to be an effective way to counter coronary micro-vasospasm that causes the slow flow phenomenon.^48,49^ The advantage of targeting T-type VGCCs is also supported by clinical trials indicating T-type block may be more effective than L-type block in cardiovascular disease.^50–52^ In part, this may be because the former have less effect against the myogenic reactivity of small arteries responsible for tissue blood flow autoregulation.

## Acknowledgments

J.F. Smith, H.A.L. Lemmey, L. Borysova, C.R. Hiley performed experiments, analyzed data, and approved the manuscript in final form. K.A. Dora contributed to study design, analyzed data, prepared figures, contributed to manuscript preparation, and approved the final manuscript. C.J. Garland conceived the study, performed the majority of experiments, analyzed, data and wrote and approved the manuscript.

## Sources of Funding

This work was supported by a Leon and Iris Beghian Graduate Scholarship, Magdalen College Oxford to J.F. Smith; H.A.L. Lemmey by a British Heart Foundation 4-year PhD studentship, FS/15/68/32042; British Heart Foundation Grant PG/14/58/30998; British Heart Foundation Senior Basic Science Fellowship to K.A. Dora, FS/13/16/30199

## Disclosures

None.

## Supplementary Material



## Novelty and Significance

### What Is New?

- Loss of endothelial NO switches on electrical excitability in small resistance arteries, including SHR arteries, enabling Ca^2+^ based smooth muscle transient spikes and vasospasm.
- Transient spikes require a trigger input from T-type voltage-gated Ca^2+^ channels (VGCCs), which adds to the activity of the smooth muscle L-type VGCCs that underpin physiological vasomotion responsible for optimizing tissue blood flow.
- When the endothelium can generate hyperpolarization (endothelium-dependent hyperpolarization), vasospasm is interspersed by transient vasorelaxation.
- Block of T-type VGCCs abolishes transient spikes and vasospasm, but not myogenic vasoconstriction, which is mediated by L-type VGCCs and necessary for blood flow autoregulation

### What Is Relevant?

- Endothelial cell dysfunction is due to compromised NO and endothelium-dependent hyperpolarization including hypertension. Linking loss of NO to the recruitment of latent T-type VGCCs and transient spike firing helps explain increased vasoreactivity responsible for pathological vasospasm.
- Blocking T-type VGCCs may offer a more effective means to treat cardiovascular disease than the widespread use of L-type blockers, particularly if the former do not compromise myogenic tone in the microcirculation.

### Summary

Loss of either endothelial NO or endothelium-dependent hyperpolarization raises the reactivity of small arteries. However, only loss of NO provokes input from T-type VGCCs leading to smooth muscle transient spikes and vasospasm. This change seems to be cGMP based. If loss of endothelium-dependent hyperpolarization is incomplete, it may limit vasospasm. T-type blockers abolished transient spikes and associated constriction in small coronary arteries but without affecting myogenic tone, which relies on L-type VGCCs.
