# Supplementary figures and images for: Endothelial Nitric Oxide Suppresses Action-Potential-Like Transient Spikes and Vasospasm in Small Resistance Arteries
Source: Hypertension. 2020 Jul 27;76(3):785–94. doi: 10.1161/HYPERTENSIONAHA.120.15491 (PMC7418934; doi:10.1161/HYPERTENSIONAHA.120.15491)

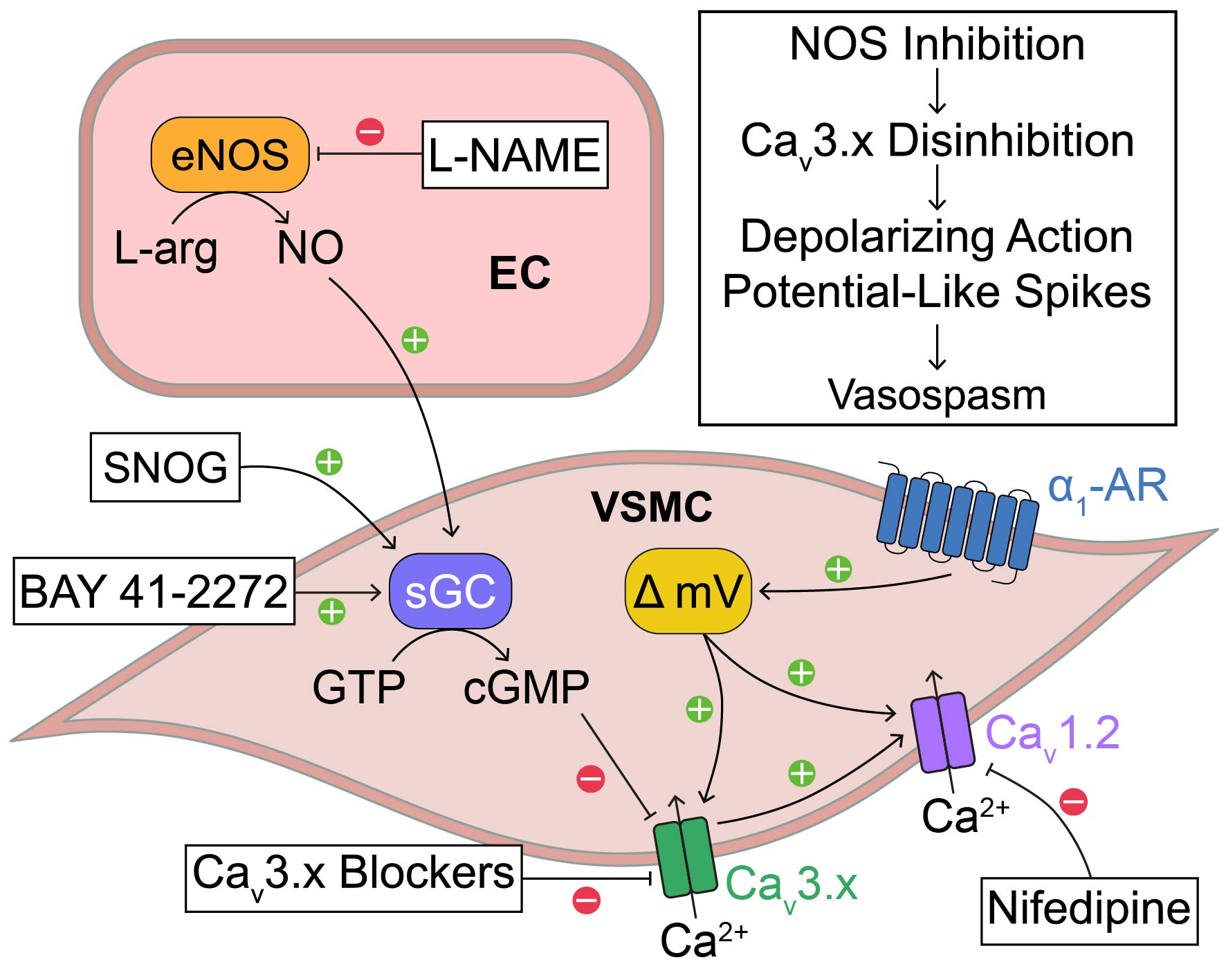

Supplement: Supplementary file 1 [file hyp-76-0785-s001.jpg]
