# Supplementary material for: Endothelial Nitric Oxide Suppresses Action-Potential-Like Transient Spikes and Vasospasm in Small Resistance Arteries
Source: Hypertension. 2020 Jul 27;76(3):785–94. doi: 10.1161/HYPERTENSIONAHA.120.15491 (PMC7418934; doi:10.1161/HYPERTENSIONAHA.120.15491)

Endothelial NO suppresses action-potential-like transient spikes and vasospasm in small resistance arteries

Josh F. Smith, Hamish A.L. Lemmey, Lyudmyla Borysova, C. Robin Hiley, Kim A. Dora & Christopher J. Garland*

*Author for correspondence:

Department of Pharmacology,
University of Oxford,
Mansfield Road,
Oxford OX1 3QT

e-mail: christopher.garland@pharm.ox.ac.uk

Short title: VGCCs and small artery vasospasm

Expanded Materials and Methods:

Rat small mesenteric and coronary arteries were isolated and mounted in a Mulvany-Halpern myograph for simultaneous measurement of tension with either membrane potential and intracellular calcium. Isolated arteries and endothelial cell tubes were prepared for RT-PCR.[1-4] Data were analyzed using Microsoft Excel 2011 (Microsoft Corporation) and GraphPad Prism (v8.0, GraphPad Software, USA) software. Data that support the findings of this study are available from the corresponding author upon reasonable request.

Animal use was approved by the University of Oxford ethical committee and complied with the Animals (Scientific Procedures) Act 1986 and European Directive 201/63/EU. Animals were housed in a temperature-controlled environment with a 24-hour light-dark cycle and water *ad libitum.* These studies comply with ARRIVE guidelines. [2]

Preparation of arteries for myography:

Male Wistar, Wistar Kyoto (WKY) or SHR rats (225-323 g) were killed as specified by Schedule 1 of the Animals (Scientific Procedures) Act 1986, UK. The mesenteric arcade was immediately removed and placed in ice-cold MOPS-buffered solution containing 145.0 mmol/L NaCl, 4.7 mmol/L KCl, 2.0 mmol/L $CaCl_2$, 1.17 mmol/L $MgSO_4$, 2.0 mmol/L MOPS, 1.2 mmol/L NaH_2 PO_4 , 5.0 mmol/L glucose, 2.0 mmol/L pyruvate, 0.02 mmol/L EDTA, and 2.75 mmol/L NaOH (pH 7.40 ± 0.02 at 37°C). A third-order mesenteric artery (external diameter between 230-300 µm at 70 mmHg) was dissected free of adherent tissue and a small segment (2 mm) removed and mounted in a Mulvany-Halpern wire myograph (model 400A, Danish Myo Technology, Denmark) in Krebs solution containing (mmol/L): 118 NaCl, 25 $NaHCO_3$, 3.6 KCl, 1.2 $MgSO_4{\cdot}7H_2O$, 1.2 KH_2PO_4, 2.5 $CaCl_2$, 11 glucose and gassed with 21% O_2, 5% CO_2, with N_2. The solution temperature was raised to 37° C, and arteries normalized to a resting tension equivalent to that generated at 90% of the diameter of the vessel at 100 mmHg. Artery reactivity was assessed by pre-constriction to phenylephrine (PE, 0.5–3 µmol/L) followed by endothelium-dependent vasorelaxation to acetylcholine (ACh, 0.1 and 1 µmol/L). Only vessels relaxing >95% were used further. Endothelial cell NO synthesis was either inhibited using 100 µmol/L L-NAME added to the Krebs buffer or by stripping the endothelial surface by rubbing with a human hair. With the latter, arteries were then remounted and loss of endothelium assessed by complete loss of vasorelaxation to 1 µmol/L ACh. As with mesenteric arteries, intra-septal coronary arteries, *circa* 2mm in length and 220 – 340µmol/L in diameter at 80 mmHg, were dissected from surrounding tissue, in this case cardiac myocytes, and placed in chilled MOPS-buffer. A section free from side-branches was identified, excised, mounted and normalized in a Mulvany-Halpern wire myograph to a resting tension equivalent to that generated at 90% of the diameter of the vessel at 80 mmHg. Reactivity was assessed by contraction with 45mmol/L K^+ Krebs and 1µmol/L U46619, then with subsequent vasorelaxation to ACh.

Measurement of smooth muscle membrane potential:

The vascular smooth muscle membrane potential was measured using sharp glass microelectrodes backfilled with 2 mol/L KCl (tip resistances *circa* 100 MΩ), as previously described.[3] Smooth muscle membrane potential was recorded through a pre-amplifier (Neurolog system, Digitimer Ltd., U.K.) linked to a MacLab data acquisition system (AD Instruments Model 4e, usually at 100 Hz).

Endothelial cell tube isolation:

Endothelial tubes were isolated as described previously. [1] Arteries were dissected in cold dissection buffer containing (in mmol/L): 137.0 NaCl, 5.6 KCl, 1.0 $MgCl_2$, 10.0 HEPES, 10.0 glucose, 0.01 sodium nitroprusside and 0.1% BSA. Second- and third-order mesenteric

arteries were dissected free of surrounding tissue. One end of the artery was cannulated onto a glass micropipette (external diameter 100–120 μm) and the lumen flushed with cold dissection buffer to remove blood. Arteries were cut into 2–3 mm segments and transferred to a clear 1.5 mL microcentrifuge tube containing 1 mL of cold dissection buffer. Artery segments were washed twice with enzyme free dissociation buffer containing (in mmol/L): 137.0 NaCl, 5.6 KCl, 1.0 $MgCl_2$, 10.0 HEPES, 10.0 glucose, 2.0 $CaCl_2$ and 0.1% BSA at 37°C. Segments were then incubated in dissociation buffer supplemented with 0.62 mg. mL^{-1} papain, 1.0 mg. mL^{-1} dithioerythritol and 1.5 mg. mL^{-1} collagenase for 20–25 min at 37°C. Enzymatic digestion was terminated by aspiration of enzyme containing buffer and replaced with enzyme free dissociation buffer in a round culture dish for trituration. EC tubes were dissociated from surrounding vascular smooth muscle cells by gentle trituration using a glass micropipette (inner diameter 80–110 μm). A nanolitre injector (Nanoliter 2010) coupled with a Micro4 controller (World Precision Instruments, Sarasota, FL, USA) was mounted on an upright Olympus BX51WI microscope to allow for real-time visualization of the trituration process.

<u>RNA extraction and quantitative RT-PCR</u>:

Arteries and EC tubes were isolated as described above. Four segments of second- and third-order artery branches from one animal were pooled for each *n* value. Arteries were homogenized and RNA was extracted using the RNeasy plus Mini Kit (Qiagen, Valancia, CA, USA). For EC tubes, three >1 mm long EC tubes were pooled from one animal for each *n* value. Isolated EC tubes were transferred using a new glass micropipette into a clean round culture dish containing enzyme free dissociation buffer, and repeated three times to reduce VSMC contamination. RNA was then extracted from EC tubes using Cells-to-CT 1-Step TaqMan Kit lysis buffer (ThermoFisher Scientific) in accordance with the manufacturer's instructions.

Quantitative RT-PCR was carried out using the Cells-to-CT 1-Step TaqMan Kit. Pre-validated, gene specific FAM-conjugated Taqman probes were purchased from ThermoFisher Scientific Table S1. Reverse transcription was performed at 50°C for 20 min, followed by heat activation of Taq polymerase at 95°C for 30 s. PCR thermocycling conditions comprised 95°C for 15 s, then 60°C for 1 min, for 40 cycles, using the 7500 Fast Real-Time PCR system (ThermoFisher Scientific). All samples were run in duplicate, and a no-template negative control was included for each gene. Relative gene expression was calculated with the D cycle threshold (DCt) method and normalized to both hypoxanthine phosphoribosyl transferase 1 (Hprt1) and b-actin (Actb) as housekeeping genes. The absence of smooth muscle cells in each tube extraction was assessed by expression of the smooth muscle cell marker α–smooth muscle actin (Acta2); in all cases, a Ct value of >36 was considered “not detected.”

<u>Measurement of VSM intracellular Ca^{2+}</u>:

Changes in intracellular $[Ca^{2+}]_i$ were imaged in a confocal myograph chamber following incubation of arteries in HEPES-buffered solution containing 2μmol/L Calbryte 520AM (AAT Bioquest) and 0.02% pluronic F-127 (Termo Fisher Scientific) for 30 min at 30°C. HEPES contained (in mmol/L): 130 NaCl, 5 KCl, 1.2 $MgCl_2$, 1 $CaCl_2$, 2 pyruvate (Na salt), 10 HEPES and 10 glucose. Arteries were then incubated in Krebs buffer for 30 min to allow de-esterification. Confocal images were obtained using a x40 (0.8 NA, Olympus) water immersion objective and iXon 887 EMCCD camera (Andor Technology, UK) coupled to a Nipkow spinning disk confocal head (CSU22, Yokogawa, Japan) and inverted microscope (IX70, Olympus, Japan). Fluorescence intensity at 513-563 nm (excitation 488 nm) was acquired from the bottom surface of arteries at 40 Hz (Andor iQ v3.5, Andor Bioimaging Division, UK). Experiments were performed in the presence of L-NAME (100μmol/L) at ~36°C. Fast Ca^{2+} events (Ca^{2+} flashes, duration at 50% < 339±11ms, n=5) synchronized between neighbouring cells were induced by phenylephrine (0.3μmol/L) and acquired from 430x420 pixels. Data are

expressed either as relative fluorescence (F/F_0) from the full field of view or the frequency of Ca^{2+} events observed per min.

Data analysis:

Data were analyzed using Microsoft Excel 2011 (Microsoft Corporation) and GraphPad Prism (v8.0, GraphPad Software, USA) software.

Vasodilation was expressed as a percentage reversal of tone induced by PE (100% corresponding to the maximal diameter). Results are summarized as mean ± S.E.M. of *n* replicates, where *n* is the number of individual arteries, each obtained from a separate animal. Statistical analyses for concentration response curves were performed by t-test, and for other data by ANOVA, each with Bonferroni-Dunn post-tests. $P < 0.05$ indicated statistical significance. Fourier transforms were performed on the raw data to provide a quantitative analysis of the waveforms resulting from various treatments. This was carried out through a proprietary MATLAB script allowing a frequency comparison of the different waveforms, as well as a visual depiction of the aggregated mean experiments per treatment and comparison of signal to noise ratio, calculated as the mean peaks of the Fourier spectra 25% above the basal frequencies, in effect giving a measurement for the symmetricity of the waveform. Changes in membrane potential, frequency, amplitude and burst frequency was assessed with proprietary software (LabChart 7).

Materials:

Drug/molecular target nomenclature follows the BJP Concise Guide to Pharmacology. [15] All drugs were obtained from Sigma (UK) with the exception of apamin (Latoxan), levcromakalim, NNC 55-0396 (both Tocris), ODQ (Calbiochem, UK), TTX (Latoxan). ODQ and TRAM-34 were prepared in DMSO. All other stock solutions were prepared using purified (MilliQ) water. All stock solutions were prepared at 10^{-1} or 10^{-2} Mol/L and subsequently diluted in Krebs buffer. Inhibitors were pre-incubated with the arterial tissue for at least 20 minutes before agonist application, with the exception of apamin (1 h). All drugs were added directly to the bath.

References for Materials and Methods:

1. Garland CJ, Bagher P, Powell C, Ye X, Lemmey HAL, Borysova L, Dora KA. Voltage-dependent Ca^{2+} entry into smooth muscle during contraction promotes endothelium-mediated feedback vasodilation in arterioles. *Sci Signal*. 2017;10:eaal3806
2. McGrath JC, Lilley E. Implementing guidelines on reporting research using animals (arrive etc.): New requirements for publication in bjp. *Br J Pharmacol*. 2015;172:3189-3193
3. Garland CJ, McPherson GA. Evidence that nitric oxide does not mediate the hyperpolarization and relaxation to acetylcholine in the rat small mesenteric artery. *Br J Pharmacol*. 1992;105:429-435
4. Alexander SP, Benson HE, Faccenda E, Pawson AJ, Sharman JL, Spedding M, Peters JA, Harmar AJ, Collaborators C. The concise guide to pharmacology 2013/14: G protein-coupled receptors. *Br J Pharmacol*. 2013;170:1459-1581

Supplementary Figures:

Figure S1: Depolarizing spike potentials induced by PE. Expanded images of a 20 second sample taken from Figure 1B (upper trace), with PE applied to a mesenteric artery in the presence of 100µM L-NAME and Figure 1C (lower trace), PE applied to a denuded mesenteric artery.

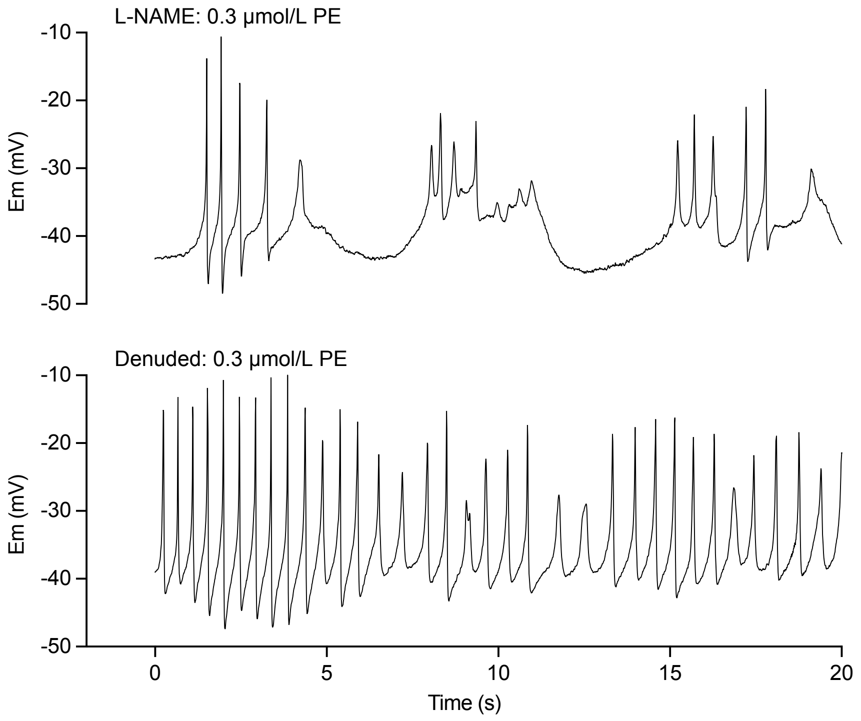

Figure S2: **Concentration-response curves showing block of T-type VGCCs reverses enhanced vasoconstriction to PE in denuded mesenteric arteries but not in the absence of EDH**

a. Denuding ECs enhanced vasoconstriction to PE (*P<0.01) an effect subsequently suppressed by 0.3μmol/L NNC 55-0396 (difference between control and NNC 55-0396, P>0.01, n=5) **b.** block of EDH with a combination of 1μmol/L TRAM-34 and 0.1μmol/L apamin enhanced PE vasoconstriction, which was not reversed by 0.3μmol/L NNC 55-0396 (*control vs NNC 55-0389 P<0.01, n=5). Each point represents the mean ± SEM.

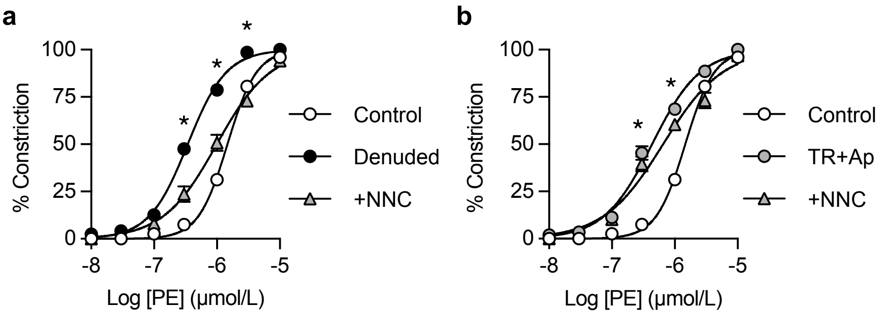

Figure S3: Original traces of membrane potential and tension showing the influence of the T-type VGCC blocker Ni^{2+} in mesenteric arteries stimulated with PE. a. vasomotion to PE persisted in the presence of 50µmol/L Ni^{2+} **b.** Ongoing PE-induced depolarizing spikes and vasoconstriction were abolished by the addition of 50µmol/L Ni^{2+}. The break in the membrane potential recording occurred when the microelectrode was dislodged. The cell was subsequently re-impaled. PE depolarized the smooth muscle to -38.9 ± 3.0mV and evoked spikes. The membrane potential was repolarized to -52.6 ± 3.0mV by Ni^{2+} with associated vasorelaxation, n=5. **c.** Depolarizing spikes and vasoconstriction to PE (left-hand traces) were abolished on washout of PE. Preincubation in 50µmol/L Ni^{2+} followed by re-stimulation with PE caused depolarization but without spike potentials (right-hand traces). Dotted lines indicate pre-stimulation membrane potential (mV) or tension (mN) levels.

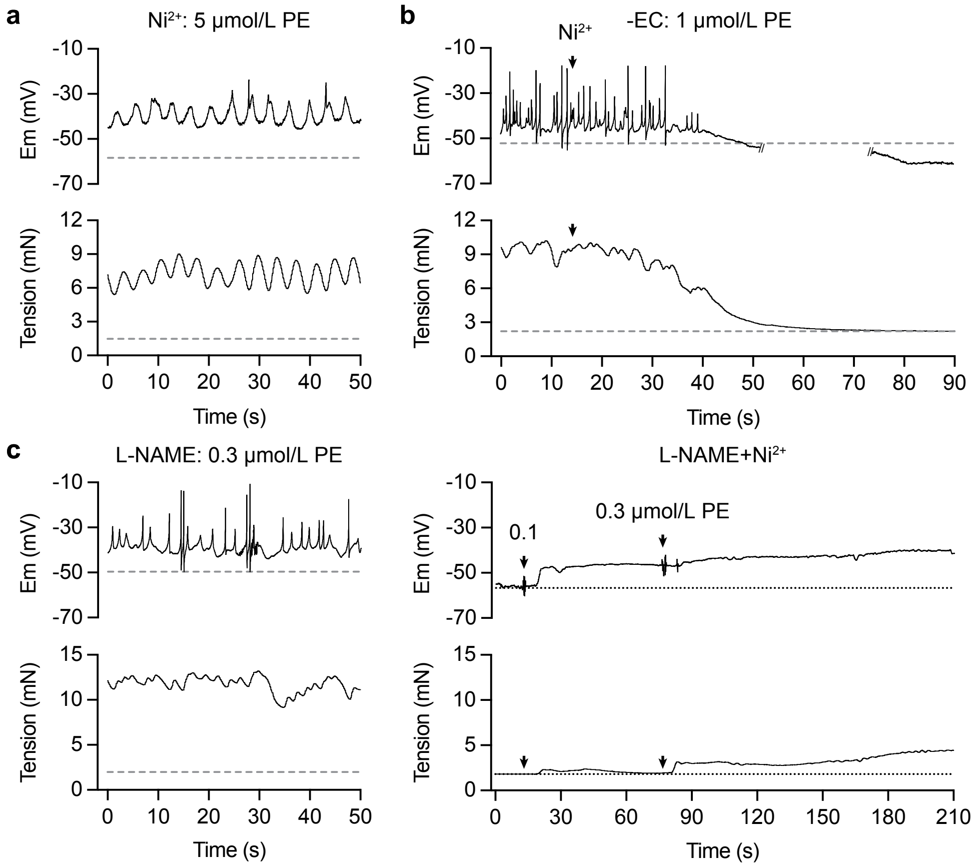

Figure S4: **Cumulative addition of T-type VGCC blockers Ni^{2+}, NNC 55-0396 or TTA-A2 during vasoconstriction of mesenteric arteries.** Each blocker reversed vasoconstriction to the L-type VGCC activator, Bay-K 8644, but only in concentrations far greater than used in the current study. Vasorelaxation to **a.** Ni^{2+}, against PE vasoconstriction in the presence of 100µmol/L L-NAME, EC_{50} 1.1µmol/L (left curve, n=5) or 70nmol/L Bay-K 8644, EC_{50} 166µmol/L, (right curve, n=6) **b.** NNC 55-0396 against 70nmol/L Bay-K 8644, EC_{50} 6.7 µmol/L (n=5) **c.** TTA-A2 against 70nmol/L Bay-K 8644, EC_{50} 8.1µmol/L (n=5). Each point, mean ± SEM.

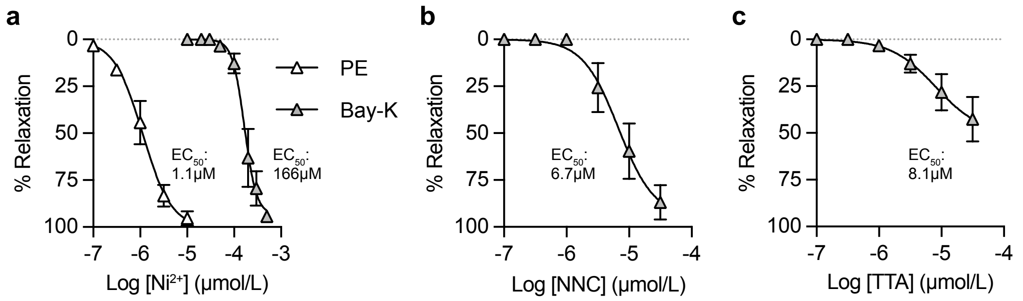

Figure S5: **The L-type (Ca_V1.2) VGCC activator, Bay-K 8644 stimulates depolarizing spike potentials and vasospasm in mesenteric arteries sensitive to L-type but not T-type VGCC blockers**. **a.** continuous spikes and vasospasm in the presence of the T-type blocker 0.3 µmol/L NNC 55-0396. **b.** mean ± SEM membrane potential and tension in denuded arteries (-EC) before (light-grey) and during exposure to 0.1µmol/L Bay-K 8644 alone and then in the presence of (0.3µmol/L NNC 55-0396 dark-grey, no effect), followed by addition of 0.3µmol/L nifedipine that abolished spikes and vasoconstriction n= 5, *P<0.001. **c.** Bay-K 8644 induced Ca^{2+} flashes and vasoconstriction in the presence of 0.3 µmol/L NNC 55-0396 that was abolished by subsequent addition of 0.3µmol/L nifedipine. *P<0.0001. **d.** 3µmol/L PE stimulated depolarization and vasoconstriction in denuded arteries without initiating spikes if applied in the presence of 0.3µmol/L nifedipine **e.** membrane potential and tension before (light-grey) and during exposure to 0.7µmol/L PE in denuded arteries (-EC), then in the same arteries after washout, incubation with 0.3 µmol/L nifedipine (Nif) and PE again when at a similar mean mV spikes were not evoked but vasoconstriction was reduced, *P<0.05, n=5. **f.** in denuded arteries membrane potential and tension before to 0.07µmol/L Bay-K 8644 (-EC), was not altered with 0.3µmol/L TTA-A2 present (+TTA), N.S. P>0.05, n=5. Boxes show mean ± SEM of maximum and minimum mV and mN, dotted lines and light-grey indicates pre-stimulation values.

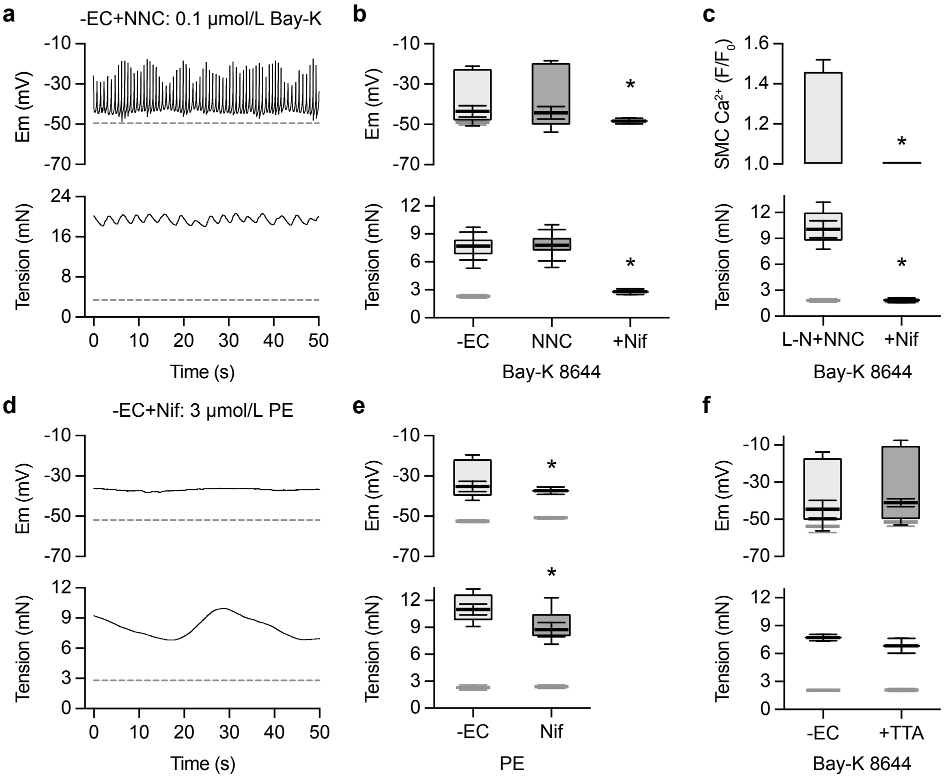

Figure S6: Vasoreactivity of Wistar, Wistar-Kyoto (WKY) and spontaneously hypertensive rat (SHR) mesenteric small arteries. a. maximum vasoconstriction stimulated by 10µmol/L PE, 1µmol/L U46619 and 45mmol/L KCl PSS. SHR arteries developed *circa* 30% more vasoconstriction than Wistar or WKY arteries *P<0.0001 and #P<0.001, respectively, n=6 in each case **b.** vasoconstriction to PE in arteries before (left panel) and during exposure to 100 µmol/L L-NAME (right panel), which raised sensitivity to PE, n=5-6 in each case. **Left panel**: 1 and 3 µmol/L vs WKY #P< 0.05 and 0.01, respectively; vs Wistar * P<0.0001 and 0.001, respectively. WKY vs Wistar 1µmol/L * P<0.01. **Right panel:** SHR vs WKY and Wistar # * both P< 0.01.

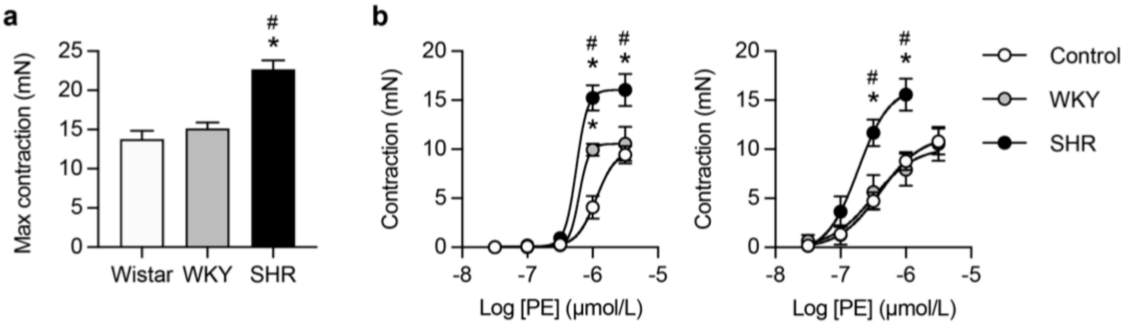

Figure S7: Small myogenic coronary arteries developed T-type VGCC-dependent depolarizing spikes and vasoconstriction after block of NO synthase
a. Depolarizing spike potentials and vasoconstriction on top of myogenic tone in the presence of 100µmol/L L-NAME. Dotted lines represent membrane potential and tension before exposure to L-NAME (myogenic tone) **b.** mean ± SEM resting membrane potential and tension before (light-grey) myogenic tone developed. Myogenic tone shown as box-plots (Control). Adding L-NAME stimulated spike firing (amplitude 12 ± 1.6mV) and further vasoconstriction, with significant change in both mean mV and mN *P<0.05 and P<0.01, n=7 & 14. Subsequent addition of 0.3µmol/L NNC 55-0396 abolished spikes without changing mean membrane potential (mV) and also reduced vasoconstriction, #P<0.01, n=11. **c.** development of myogenic tone (control) and then the addition of 100µM L-NAME correlate with progressive smooth muscle depolarization, r^2= 0.579, P<0.0001. Individual data points for separate experiments from coronary arteries without myogenic tone shown as white circles (at 0mN), mean ± SEM mN and mV white boxes, n =5. Arteries with myogenic tone (Control and mean ± SEM as box, n=8) and following 100µmol/L L-NAME (dark-grey circles and box showing mean ± SEM, n= 10).

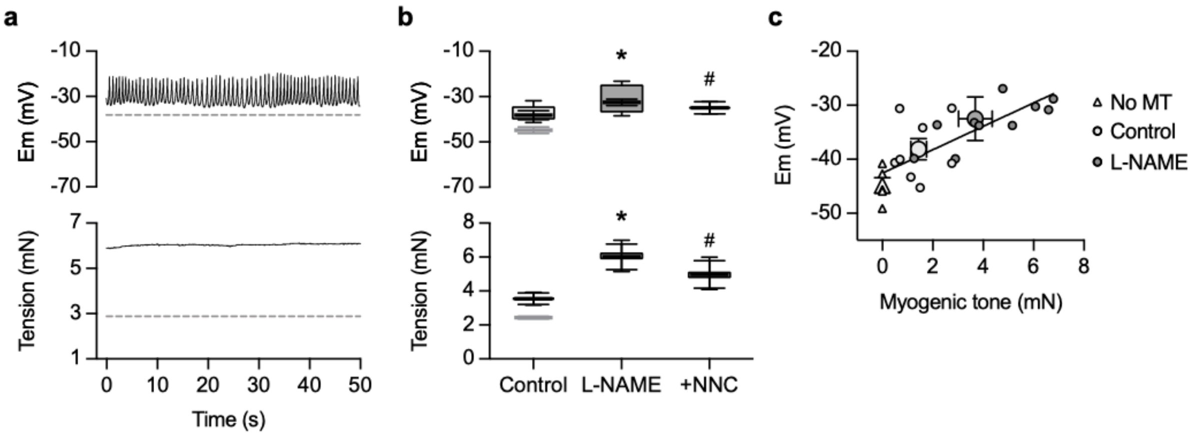

Figure S8: Summary of interaction between endothelial (EC) and vascular smooth muscle cells (SMC). Increase in EC Ca^{2+} follows an increase in SMC Ca^{2+} and serves to increase nitric oxide synthase (NOS) and K_{Ca} channel activity. NO acts via sGC and cGMP to suppress T-type VGCC input and also limits L-type VGCC input to vasomotion, with hyperpolarization dropping L-type VGCC Ca^{2+} influx and thus Ca^{2+} signal to ECs. This allows depolarization to predominate again and the cycle repeats. Removing NO either by block of NOS or sGC recruits T-type VGCCs enabling depolarizing spike potentials to be triggered by phenylephrine (or spontaneously in myogenic coronary arteries). The consequence is loss of vasomotion and increased vasoreactivity with cells developing sustained vasospasm. If EDH is still functional, vasospasm is interrupted by brief periods of vasorelaxation. If NOS is blocked, vasomotion can be restored by activation of sGC and effect this appears to rely on cGMP.

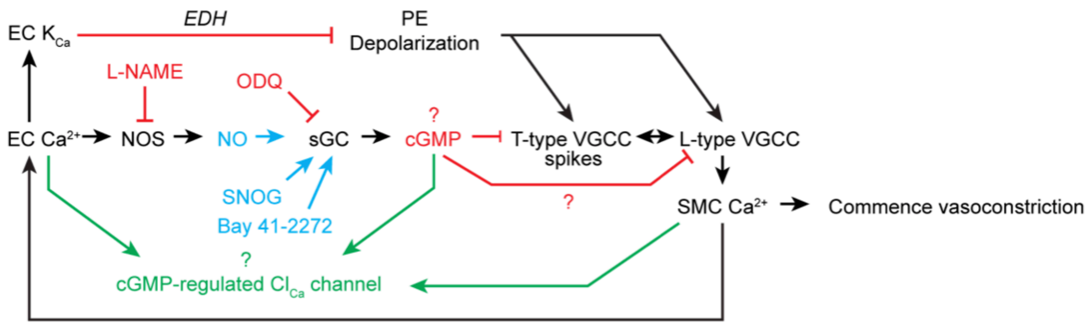

Supplement: Supplementary file 2 [file hyp-76-0785-s002.pdf]
