# Supplementary material for: Endothelial Nitric Oxide Suppresses Action-Potential-Like Transient Spikes and Vasospasm in Small Resistance Arteries
Source: Hypertension. 2020 Jul 27;76(3):785–94. doi: 10.1161/HYPERTENSIONAHA.120.15491 (PMC7418934; doi:10.1161/HYPERTENSIONAHA.120.15491)
Supplement: Supplementary file 3 [file hyp-76-0785-s003.pdf]

*** Short In Vivo Checklist**
AHA - Preclinical Animal Testing: Prevention of bias is important for experimental cardiovascular research. ***This short checklist must be completed, and the answers should be clearly presented in the manuscript as well.*** *The checklist will be used by reviewers and editors but will not be published. If a revision is requested, you will be required to complete at revision submission a more detailed checklist that will be published with the accepted article.*

This study involves animals:
Yes

Animals

Species, age, sex, strains, and sources of animals are described:	Yes

Randomization

Randomization and allocation concealment were performed:	No

Blinding

Blinding was performed:	Yes

Inclusions and Exclusions (a)

Specific criteria for inclusions and exclusions are specified:	Yes

Inclusions and Exclusions (b)

Criteria for inclusions and exclusions were set before the study:	Yes

Reporting of Excluded Animals

All animals excluded after the randomization are reported:	No

Statistical Methods

Statistical Methods are described:	Yes

Date completed: 06/17/2020 10:14:34
User pid: 46325
